# Antitumour effects of apatinib in progressive, metastatic differentiated thyroid cancer (DTC)

**DOI:** 10.1007/s12020-022-03113-9

**Published:** 2022-06-29

**Authors:** Liang Shi, Qinqin You, Jun Wang, Hanjin Wang, Shaohua Li, Rui Tian, Xiaocheng Yao, Wenyu Wu, Lele Zhang, Feng Wang, Yansong Lin, Shuren Li

**Affiliations:** 1 Department of Nuclear Medicine, Nanjing First Hospital, Nanjing Medical University, Nanjing, China; 2 Department of General Surgery, Nanjing First Hospital, Nanjing Medical University, Nanjing, China; 3 Department of Nuclear Medicine, Peking Union Medical College (PUMC) Hospital, Chinese Academy of Medical Sciences & PUMC, Beijing, China; 4 Division of Nuclear Medicine, Department of Biomedical Imaging and Image-guided Therapy, Medical University of Vienna, Vienna, Austria

**Keywords:** Metastatic differentiated thyroid cancer, Apatinib, Tyrosine kinase inhibitor, Radioactive iodine (RAI)

## Abstract

**Purpose:**

Management of progressive, metastatic radioactive iodine refractory differentiated thyroid cancer (RAIR-DTC) has been a great challenge due to its poor prognosis and limited treatment options. Recently, apatinib, an orally anti-angiogenic tyrosine kinase inhibitor (TKI) is reported to be useful for treatment of progressive RAIR-DIC. The aim of this study was to evaluate the antitumour effect of apatinib and the combination therapy with radioactive iodine (RAI) in patients with progressive metastatic DTC.

**Methods:**

Five patients (all female, mean age 62 ± 8 years, ranged from 51 to 69 years) with distant metastatic DTC (dmDTC) after total thyroidectomy (TTE) and neck lymph node dissection were treated with apatinib at a dose 500 mg per day after ^18^F-Fluorodeoxyglucose (^18^F-FDG) PET/CT. The effects of apatinib on DTC were evaluated at 4 ± 1 months after treatment with apatinib. RAI therapy was then initiated. The response to apatinib and the combination therapy with RAI treatment was evaluated by Response Evaluation Criteria in Solid Tumours (RECIST, version 1.1) and metabolic activity using serum thyroglobulin (Tg) and ^18^F-FDG PET/CT.

**Results:**

Positive ^18^F-FDG PET/CT results were found in all patients before apatinib therapy. The immunohistochemical analysis of primary tumour tissues showed high expression of vascular endothelial growth factor receptor-2 (VEGFR-2). Four patients with follicular thyroid carcinoma (FTC) showed partial response (PR) with significant decrease in tumour size and maximum standardized uptake value (SUVmax) after 4 ± 1 month’s treatment with apatinib. Further significant reduction of tumour size and SUVmax were observed in three patients after combination therapy with apatinib and RAI. Only one patient with both FTC and papillary thyroid cancer (PTC) demonstrated progressive disease (PD) after treatment with apatinib alone, however, a decrease in tumour size and SUVmax as well as serum Tg levels was achieved after the combination with RAI therapy and apatinib.

**Conclusions:**

Apatinib had significant antitumour effects on progressive distant metastatic DTC. Moreover, beneficial synergistic and complementary effects were shown when apatinib combined with RAI therapy.

**Clinical Trial Registration:**

NCT 04180007, Registered November 26, 2019.

## Introduction

Approximately 10–20% of differentiated thyroid cancer (DTC) patients have progress distant metastatic DTC (dmDTC) [1, 2]. A part of them belongs to radioactive iodine refractory DTC (RAIR-DTC) [1, 2]. The RAIR-DTC is leading cause of thyroid cancer related death [3]. The dmDTC presents a major management challenge. FDA has approved two agents targeting vascular endothelial growth factor receptors (VEGFRs), lenvatinib and sorafenib for treatment of RAIR-DTC [4, 5]. Although sorafenib and lenvatinib were found to prolong progression-free survival (PFS) in patients with RAIR-DTC, however, no significant benefit on overall survival (OS) was observed, except for a subgroup of patients older than 65 years in the SELECT trial [4, 5]. Therefore, alternative treatment options are needed for patients with RAIR-DTC. Recently, our study showed that apatinib, a small-molecule tyrosine kinase inhibitor (TKI) targeting vascular endothelial growth factor receptor 2 (VEGFR2) and platelet-derived growth factor receptor (PDGFR) β [6], demonstrates significant clinical benefits in both prolonged PFS and OS in patients with progressive locally advanced or metastatic RAIR-DTC [7, 8]. In this preliminary study, we want to evaluate the role of apatinib on dmDTC before RAI and the effects of combination therapy with RAI and apatinib.

## Material and methods

### Patients

This study was conducted in accordance with the Declaration of Helsinki and International Conference on Harmonization Good Clinical Practice guidelines and approved by the Ethics Committee of Nanjing First Hospital. Written informed consent was obtained from all patients.

Five patients, who had total thyroidectomy (TTE) and neck lymph node dissection before the study and at least one ^18^F-Fluorodeoxyglucose (^18^F-FDG)-avid distant metastatic lesion as well as no previous RAI or TKI therapy, were included in the study (Table 1).

**Table 1:** Characteristics of patients and results

No	Age	Gender	Type	TNM	Dominant metastasis site	Apatinib dosis (mg/day)	Accumulated RAI Dose (GBq)	post therapeutic ^131^I scan	BaselineTg (ng/mL)	Tg after apatinib (ng/mL)	Tg after apatinib + RAI (ng/mL)	Baseline SUVmax	SUVmax after apatinib	SUVmax after Apatinib+RAI	Baseline tumour size (cm)	tumour size after apatinib (cm)	tumour size after apatinib + RAI (cm)	tumour size decresae after apatinib	tumour size decresae after apatinib + RAI	Response^a^ after apatinib	Response^a^ after apatinib + RAI
1	64	F	FTC	T1bN0M1	10th right rib	500	33.30	positive	8644	887	6	10,93	4,57	3,12	1,5	1,2	0,6	31%	81%	PR	PR
					left acetabulum			positive				11,52	8,47	4,61	4,4	2,9	0,5				
2	69	F	FTC	T3bN0M1	left os ischii	500	16.65	positive	12470	3346	8	7,46	4,82	4,15	5,4	3,8	2,1	30%	61%	PR	PR
3	68	F	FTC	T1bN0M1	left humerus	500	9.25	positive	4581	4341	2448	15,56	6,45	5,11	6,0	4,1	4,0	35%	37%	PR	PR
					Th12			positive				10,45	5,78	4,45	4,9	3,4	3,3				
					right lung			negative				4,43	3,14	2,43	1,3	0,7	0,6				
					left lung			negative				3,24	2,79	1,81	1,4	0,7	0,6				
4	51	F	FTC	TxN0M1	left 5th to 9th ribs	500	9.25	negative	74465	4439	4700	28,70	22,04	4,46	13,5	9,0	9,0	32%	35%	PR	PR
					sacrum			negative				8,94	8,50	4,13	4,2	4,0	4,0				
					liver			negative				8,21	11,28	5,77	7,0	3,5	3,1				
					mediastinum lymph node			negative				19,99	11,99	2,86	2,9	1,9	1,6				
					left pleura			negative				13,84	8,23	3,41	2,8	2,2	2,1				
5	58	F	FTC, PTC	T4aNxM1	5th rib	500	7.40	positive	20130	24480	9123	4,40	5,93	2,60	2,1	2,1	2,0	0%	5%	PD	SD
					right acetabulum			positive							2,2	3.0	2.0	(+36%)	9%		
means ± SD									24058 ± 28755	7499 ± 9600	3257 ± 3816	11,36 ± 9,47	8,00 ± 5,07^b^	3,76 ± 1,16^c,d^	4,3 ± 3,2	3,0 ± 2,2^e^	2,6 ± 2,3^f,g^				

^a^: Tumour response was evaluated according to RECIST (version 1.1); *PR* partial response, *PD* progressive disease, *SD* stable disease

^b^: *p* < 0.01 versus baseline SUVmax

^c^: *p* < 0.01 versus baseline SUVmax

^d^: *p* < 0.01 versus SUVmax after apatinib

^e^: *p* < 0.01 versus baseline tumour size

^f^: *p* < 0.01 versus baseline tumour size

^g^: *p* < 0.01 versus tumour size after apatinib

### Treatment with apatinib and RAI with radioactive iodine-131 (^131^I)

500 mg apatinib (Hengrui Medicine, Jiangsu, China) was administrated orally once daily until intolerable toxic effects occurred. Adverse events (AEs) were graded according to National Cancer Institute Common Terminology Criteria for Adverse Events (AEs) version 4.0 (CTCAE 4.0). All AEs were assessed every 4 weeks after apatinib administration. According to institutional guidelines at the time of treatment, median administered RAI activity was 9.25 GBq [200 mCi] (minimum–maximum: 7.40–8.33 GBq (200–250 mCi)) per each ^**131**^**I** treatment. The Patients were treated with ^131^I every 6–12 months according to decision of the Multidisciplinary Team (Table 2).

**Table 2:** Therapy and adverse events in patients with dmDTC

Patient	Duration of apatinib therapy (=Follow-up duration)	Number of RAI therapy	Response Evaluation (3 months after RAI + apatinib therapy)	Adverse events					
				apatinib alone			RAI + apatinib		
No.	(months)			grade 1	grade 2	grade 3	grade 1	grade 2	grade 3
1	52	4	81% decrease of tumour size	+	+	–	+	+	–
2	29	2	61% decrease of tumour size	+	+	+	+	+	+
3	18	1	37% decrease of tumour size	+	+	–	+	+	–
4	24	1	35% decrease of tumour size	+	+	–	+	+	–
5	10	1	5% decrease of tumour size	+	+	+	+	+	+

*RA*I radioactive iodine, +: adverse event positive; −: no adverse event

### ^18^F-FDG PET/CT imaging and response evaluation

**Fig. 1:**
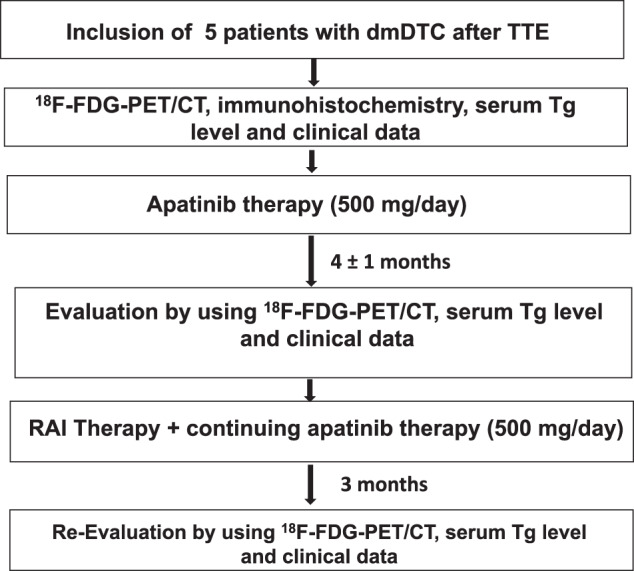
Patient flow diagram

PET imaging from the top skull to mid-thigh was performed 60 ± 5 min post intravenous injection of 3.7 MBq ^18^F-FDG per kg bodyweight. Patients fasted at least six hours before ^18^F-FDG injection. Before the ^18^F-FDG injection, the fasting blood glucose level was less than 9 mmol/L. All scans were performed on United Imaging, U780 PET/CT (Shanghai, China). Follow-up ^18^F-FDG PET/CT was performed at 4 ± 1 months after apatinib initiation and 3 months after combination therapy of apatinib and RAI treatment. Tumour response was evaluated according to RECIST (version 1.1) [9]. Serum thyroglobulin (Tg) and anti-thyroglobulin antibody (Anti-Tg Ab) level were measured at the baseline, at 4 ± 1 months after apatinib therapy and at 3 months after RAI treatment, respectively. Figure 1 showed the flow diagram of this study. Follow-up was performed at the beginning of apatinib therapy.

### Statistical analysis

All continuous variables are presented as mean ± standard deviation and were analyzed statistically using SPSS 23 software (SPSS, Dallas, TX, USA). Two-tailed paired sample *t*-tests were used to compare the tumour diameters and metabolic parameters before and after apatinib or RAI treatment. *p* < 0.05 were considered as statistically significant.

## Results

### Case 1

**Fig. 2:**
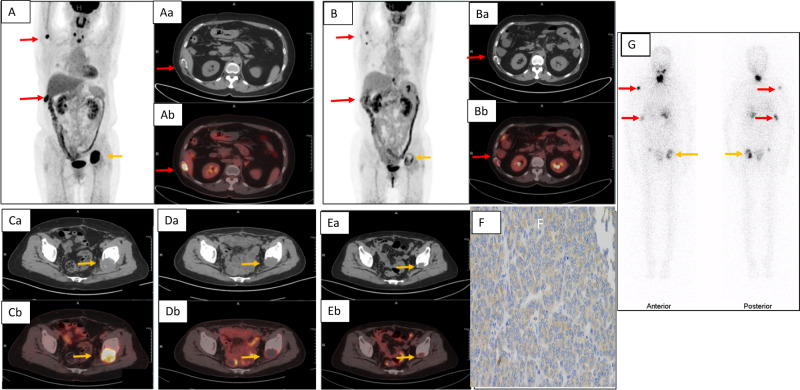
In case 1, ^18^F-FDG PET/CT showed dmDTC before apatinib therapy (**A**, **Aa**, **Ab**, **Ca** and **Cb**). Decreased tumour sizes and SUVmax in the right 10^th^ rib (red arrows) and in the left acetabulum (orange arrows) after neoadjuvant therapy with apatinib for 4 months (**B**, **Ba**, **Bb**, **Da** and **Db**) and further decrease in tumour size in the left acetabulum after apatinib combined with RAI (**Ea** and **Eb**). Immunohistochemistry assay confirmed expression of VEGFR2 in the tumour tissue (Magnification, ×400) (**F**). The post-therapeutic ^131^I whole-body scans in anterior and posterior views were shown (**G**)

A 64-year-old woman (No 1 in the Table 1) presented with pain in her left hip and right ribs with an increased serum Tg level of 8644 ng/mL. ^18^F-FDG PET/CT showed high uptake in the thyroid masses and multiple lytic bone lesions on the right humerus, 10^th^ right rib, and the left acetabulum accompanied by a large tumour infiltration (Fig. 2A, Aa, Ab, Ca and Cb). Follicular thyroid carcinoma (FTC) was diagnosed after a total thyroidectomy. High expression of VEGFR-2 was confirmed in immunohistochemical (IHC) analysis (Fig. 2F). Four months after apatinib treatment, ^18^F^-^FDG PET/CT showed remarkable decrease in tumour size and ^18^F-FDG uptake in metastases (Fig. 2B, Ba, Bb, Da, Db). Serum Tg level decreased to 887 ng/mL. Four cycles of RAI therapy were then performed with a total dose of 33,3 GBq (900 mCi) ^131^I. Post therapeutic ^131^I whole-body image showed radioactive iodine uptake in the bone metastases (Fig. 2G). Follow-up ^18^FDG-PET/CT showed clearly further decrease in tumour size and reduced FDG uptake in bone lesions (Fig. 2Ea and Eb). The serum Tg level reduced to 6 ng/mL (Table 1).

### Case 2

**Fig. 3:**
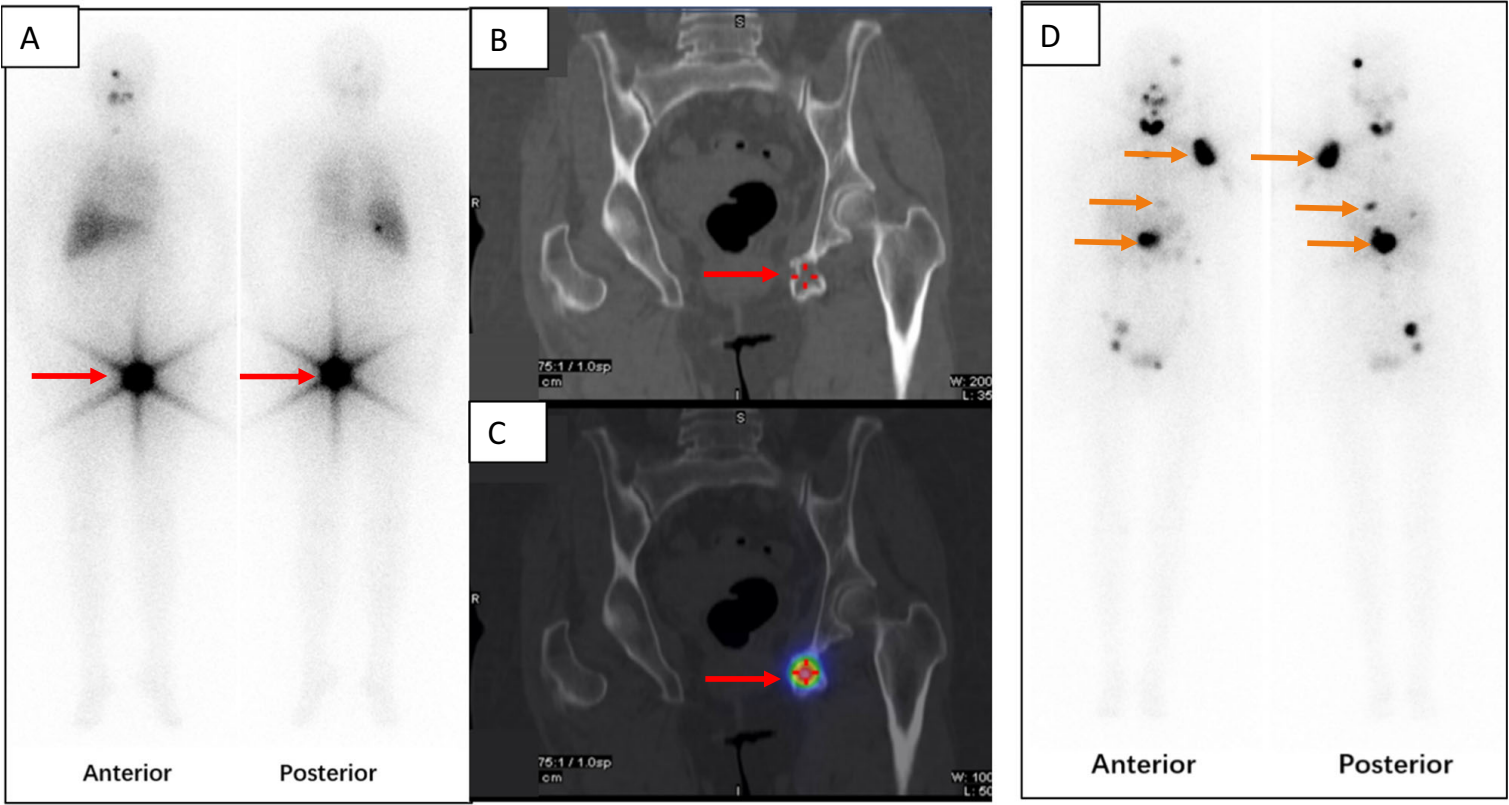
The post-therapeutic ^131^I whole-body scans of patient (No. 2 in Table 1) in anterior and posterior views (A) as well as CT (B) and SPECT/CT (C) of pelvic bone were shown. Red arrows indicated metastases. A post-therapeutic ^131^I whole-body scans of another patient (No. 3 in Table 1) in anterior and posterior views (D) were demonstrated. Orange arrows indicated metastases

A 69-year-old woman (No 2 in Table 1) had severe pain in her left hip. ^18^F-FDG PET/CT showed ^18^F-FDG-avid bone lytic lesion and tumour infiltration in the left ischium with serum Tg level of 12470 ng/mL. High expression of VEGFR-2 was confirmed with IHC analysis of the primary FTC after total thyroidectomy. Only three months after apatinib treatment, serum Tg level decreased to 3346 ng/mL. ^18^F-FDG PET/CT showed markedly reduced metastatic tumour masses with reduced FDG uptake. After two cycles of RAI therapy with a total dose of 16.65 GBq (450 mCi) ^131^I. Further tumour size decrease and metabolic activity reduction were demonstrated and Tg level decreased to 8 ng/mL. The post-therapeutic ^131^I whole-body image showed iodine-avid bone metastases (Fig. 3A–C).

### Case 3

After TTE and neck lymph node dissection, a 68-year-old woman (No 3 in Table 1) with histologically verified FTC underwent ^18^F-FDG PET/CT, which showed higher uptake in multiple bone metastases (9^th^ left posterior rib, Th11, Th12, L1 vertebras with spinal cord compression, and left humerus) and multiple pulmonary metastases. The serum Tg level was 4581 ng/mL. Rapid regression of lung metastases and bone metastases with decreased Tg level to 4341 ng/mL were observed only three months after apatinib treatment. After one cycle of RAI therapy with 9.25 GBq (250 mCi) ^131^I. Tg level decreased further to 2482 ng/mL. The post-therapeutic ^131^I whole-body image showed positive bone metastases (Fig. 3D).

### Case 4

**Fig. 4:**
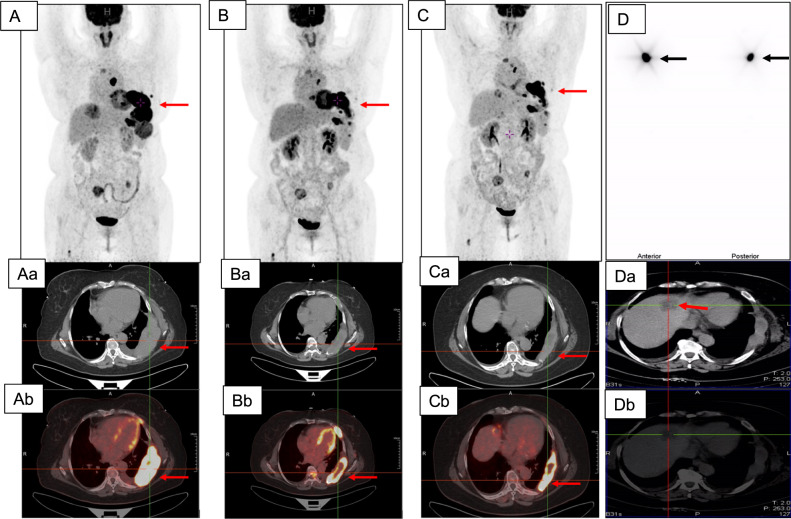
In case 4, ^18^F-FDG PET/CT revealed multiple mediastinal lymph nodes, pulmonary, liver and left 5^th^ to 9^th^ ribs as well as left thoracic wall metastases (A, Aa and Ab). Decreased tumour size and SUVmax after 4-month therapy with apatinib (B, Ba and Bb). However, no further decrease of tumour size was observed after apatinib therapy combined with RAI (**C**, **Ca** and **Cb**). Red arrows indicated metastases in left 5^th^ to 9^th^ ribs as well as left thoracic wall. The negative post-therapeutic ^131^I whole body scans in anterior and posterior views (**D**) and CT (Da) as well as SPECT/CT (Db) of liver metastasis were shown. The black arrows in D indicated thyroid remnant

11 years after TTE and neck lymph node dissection, a 51-year-old woman (No 4 in Table 1) with FTC had an increasing serum Tg level of 74465 ng/mL. Positive ^18^F-FDG PET/CT results were revealed in multiple mediastinal lymph nodes, bone (sternum, the 3rd and the 7th thoracic vertebra, the left 5^th^ to 9^th^ rib, bilateral iliac crest, and sacrum), pulmonary, liver and thoracic wall metastases (Fig. 4A, Aa and Ab). After 4 months treatment with Apatinib, the serum Tg decreased to 4439 ng/mL. ^18^F-FDG PET/CT showed notable reduction in both ^18^F-FDG uptake and tumour size of all metastatic lesions (Fig. 4B, Ba and Bb). However, three months after one cycle of RAI therapy with 9.25 GBq (250 mCi) ^131^I, no further regression of metastases and reduction of metabolic activities were observed (Fig. 4C, Ca and Cb). Tg level was constant at 4700 ng/mL. All metastases were negative in the post-therapeutic ^131^I whole-body image (Fig. 4D).

### Case 5

**Fig. 5:**
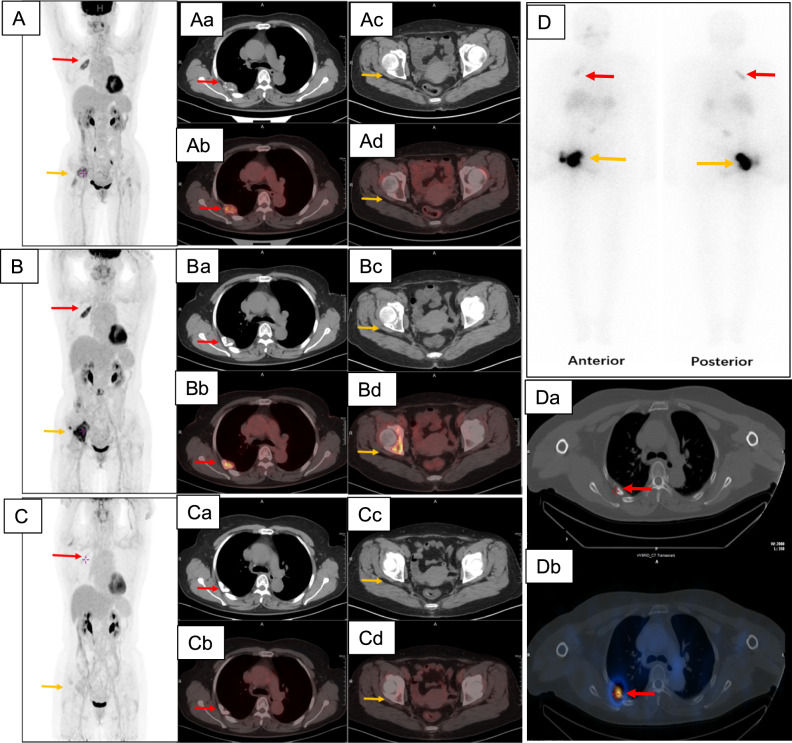
In case 5, ^18^F-FDG PET/CT showed bone (right 5^th^ rib and right acetabulum) metastases before apatinib therapy (A and Aa to Ad). Increased tumour sizes and a new metastasis formation in the right ilium were revealed after 6-month therapy with apatinib (B, Ba to Bd). After combined therapy with RAI, decreases in tumour sizes and SUVmax were shown (C, Ca to Cd). The post-therapeutic ^131^I whole body scans in anterior and posterior views (D) and CT (Da) as well as SPECT/CT (Db) of 5^th^ rib metastases were shown. Red arrows indicated metastasis in the right 5^th^ rib and orange arrows metastases in the right acetabulum

A 58-year-old woman (No 5 in the Table) suffered a lumbar pathological fracture with a cold nodule in the thyroid and increased serum Tg level (20130 ng/mL). TTE was performed and histological examinations confirmed both FTC and papillary thyroid cancer (PTC) with bone metastases. ^18^F-FDG PET/CT showed FDG-avid multiple bone metastases (Fig. 5A, Aa to Ad). Six months after apatinib therapy, the serum Tg increased to 24480 ng/mL and ^18^FDG-PET/CT showed increased tumour size and SUVmax in right acetabulum with a new bone metastasis formation in right os ilium (Fig. 5B, Ba to Bd). RAI therapy with 7.4 GBq (200 mCi) ^131^I was then performed. Post-therapeutic ^131^I-whole-body scan (WBS) showed uptake of ^131^I in bone metastases (Fig. 5D). ^18^FDG-PET/CT revealed a reduction in tumour size and SUVmax (Fig. 5C, Ca to Cd) as well as a decrease in Tg level to 9123 ng/mL in 3 months after the combination therapy with RAI and apatinib.

As shown in Table 1, statistically significant decreases in tumour size and SUVmax (both *p* < 0.01) were found in cases 1 to 4 after apatinib treatment compared with those before the apatinib therapy. Further significant reductions in tumour size and SUVmax (both *p* < 0.01) were demonstrated in cases 1, 2 and 3 after the combination therapy with RAI and apatinib compared with those after the apatinib therapy. The serum Tg levels showed remarkably reduced after apatinib therapy or after the combination with RAI, however, no statistical significance were observed due to the small numbers of patients. The anti-Tg-antibody values in all patients before and after therapy were under the reference range (≤4.11 IU/ml).

Positive post-therapeutic ^131^I whole-body images were shown in cases 1, 2, 3 and 5. All metastases were negative in the post-therapeutic ^131^I whole-body image in case 4.

All patients suffered from apatinib treatment-related AEs. The most common AEs were hand–foot skin reaction (which worsened after thyroxine withdrawal), hypertension, fatigue and proteinuria. Cases of 1, 3 and 4 had only grade 1 to 2 AEs. Only two patients (No. 2 and No. 5) had grade 3 AEs (hand–foot reaction and hypertension) for a short time (2 weeks). There were no severe AEs during and after treatment with apatinib. Apatinib combined with iodine therapy did not cause more ≥ grade 3 side effects than apatinib alone (Table 2).

The median follow-up time were 24 months (ranged from 10 to 52 months, Table 2). All patents were still alive after a year treatment with apatinib.

## Discussion

Apatinib is an orally anti-angiogenic TKI targeting VEGFR2 and PDGFR β [6] and has been approved by the National Medical Products Administration (NMPA) for advanced gastric cancer as third-line systemic therapy [10]. Recently, apatinib has been shown to be a promising treatment option for progressive locally advanced or metastatic RAIR-DTC [7, 8]. Here, we reported the beneficial antitumour effects of apatinib alone or in combination with RAI therapy on progressive dmDTC. To the best of our knowledge, this is the first investigation into the neoadjuvant therapy effects of apatinib and the combination therapy with RAI on progressive dmDTC. Our results suggested that apatinib induced significant decrease in tumour size in patients with aggressive dmDTC. Apatinib alone has antitumour effect, and beneficial synergistic and complementary effects were shown when apatinib combined with RAI therapy in the treatment of dmDTC.

Patients with dmDTC usually have an unfavorable prognosis, because some of dmDTC are RAIR-DTC [2, 3]. Furthermore, in our present study, all patients had FTC. Previous studies [11, 12] demonstrate that patients with aggressive variants of FTC have an unfavorable prognosis and exhibit lymph node or distant metastases. In many cases, these aggressive variants of thyroid carcinoma require more aggressive or innovative forms of clinical management. TKIs such as lenvatinib and sorafenib have been approved by FDA and European Medicine Agency (EMA) for progressive, metastatic RAIR-DTC [4, 5]. However, the therapy options for RAIR-DTC patients in China are still very limited due to limited availability of lenvatinib and sorafenib. TKI has been reported as a successful neoadjuvant for total thyroidectomy to reduce tumor burden [13] and enhance RAI sensitivity of thyroid cancer including an increased sodium/iodide symporter expression [14]. ^18^F-FDG PET/CT has been widely used in the diagnosis of dmDTC and ^18^F-FDG-avid lesions are usually more aggressive with poor prognosis [15, 16]. Previous studies have shown less effective of high-dose RAI therapy in patients with FDG-avid DTC than in patients with non-FDG-avid DTC [16] and there is an inverse relationship between RAI and ^18^F-FDG accumulation in DTC cells [17]. In our present study, all patients have ^18^F-FDG-avid dmDTC lesions suggesting aggressive diseases and less effective RAI therapy. We therefore explore the use of neoadjuvant treatment with apatinib prior to RAI therapy for evaluation of effect of apatinib on tumour progression and possible improvement of following RAI treatment results. Our present study demonstrated interestingly the effective treatment of apatinib alone on progressive dmDTC as shown in cases 1, 2, 3 and 4. Further decreases in tumour size and SUVmax, as well as serum Tg level, were found in combination with apatinib and RAI therapy in cases 1, 2 and 3. This might be explained by the synergistic antitumour effects of combination therapy with apatinib and RAI, since TKI has been reported to be able to enhance the RAI sensitivity of thyroid cancer due to the enhancement of sodium iodide symporter (NIS) function [14]. However, it could be also possible that this effect was only due to the longer duration of apatinib therapy. In case 4 the patient was effectively treated by apatinib, however, RAI therapy showed no effect on the dmDTC, since all metastatic lesions, in this case, were negative in post-therapeutic ^131^I scan, these metastatic lesions may be defined as RAIR-DTC. The reasons why the patient in case 5 showed no response to apatinib remain unclear. In contrast to other four patients with FTC in cases 1 to 4, this patient had both FTC and PTC. It is well known that DTC may have heterogeneous response to TKIs [18]. Apatinib suppresses tumour progression via blocking the VEGFR2 cascade in malignant cells [6]. Unfortunately, the tumour tissue VEGFR2 status, in this case, was unknown. We can only speculate that the tumour progression in this case might be through other molecular pathways rather than VEGFR2 pathway. Fortunately, this patient had response to RAI therapy leading to stable disease (SD) after RAI therapy. It might be possible that the positive effect of RAI on the metastatic lesions in case 5 might be partly due to the apatinib therapy since TKI may enhance the RAI sensitivity of thyroid cancer [14]. Our results may have implication for the complementary effect of combination therapy with apatinib and RAI as shown in cases 4 and 5.

Despite the small number of patients, our results demonstrated statistically significant reduction of tumour size and SUVmax under apatinib therapy indicating the clinical potential of apatinib therapy. Further significant decrease in tumour size and SUVmax demonstrated the synergistic and complementary effects of the combination of RAI with apatinib. The serum Tg levels which may indicate the tumour activity showed remarkably reduced after apatinib therapy and after the combination therapy with RAI, however, no statistical significance were observed due to the small numbers of patients.

There were some limitations in this study. Firstly, the number of investigated cases was limited. Secondly, there was no control group. The present study was a pilot study to evaluate the effects of apatinib on progressive dmDTC. More patients should be included in a randomized study to verify the results. Meanwhile, all patients are still alive.

**In conclusion**, apatinib is effective to inhibit the tumour progression of dmDTC. Furthermore, apatinib combined with RAI therapy might have beneficial synergistic or complementary antitumour effect on progressive dmDTC.

## Data Availability

The datasets used and/or analyzed during the current study are available from the corresponding author on reasonable request
